# The complete mitochondrial genomes of five critical phytopathogenic *Bipolaris* species: features, evolution, and phylogeny

**DOI:** 10.1186/s43008-024-00149-6

**Published:** 2024-06-11

**Authors:** Xinzheng Song, Yuehua Geng, Chao Xu, Jiaxin Li, Yashuang Guo, Yan Shi, Qingzhou Ma, Qiang Li, Meng Zhang

**Affiliations:** 1 Department of Plant Pathology, Henan Agricultural University, Zhengzhou, Henan China; 2 School of Food and Biological Engineering, Chengdu University, Chengdu, Sichuan China

**Keywords:** *Bipolaris*, Mitogenome, Intron, Gene rearrangement, Comparative analysis, Phylogenetic analysis

## Abstract

In the present study, three mitogenomes from the *Bipolaris* genus (*Bipolaris maydis*, *B. zeicola*, and *B. oryzae*) were assembled and compared with the other two reported *Bipolaris* mitogenomes (*B. oryzae* and *B. sorokiniana*). The five mitogenomes were all circular DNA molecules, with lengths ranging from 106,403 bp to 135,790 bp. The mitogenomes of the five *Bipolaris* species mainly comprised the same set of 13 core protein-coding genes (PCGs), two rRNAs, and a certain number of tRNAs and unidentified open reading frames (ORFs). The PCG length, AT skew and GC skew showed large variability among the 13 PCGs in the five mitogenomes. Across the 13 core PCGs tested, *nad6* had the least genetic distance among the 16 *Pleosporales* species we investigated, indicating that this gene was highly conserved. In addition, the *Ka/Ks* values for all 12 core PCGs (excluding *rps3*) were < 1, suggesting that these genes were subject to purifying selection. Comparative mitogenomic analyses indicate that introns were the main factor contributing to the size variation of *Bipolaris* mitogenomes. The introns of the *cox1* gene experienced frequent gain/loss events in *Pleosporales* species. The gene arrangement and collinearity in the mitogenomes of the five *Bipolaris* species were almost highly conserved within the genus. Phylogenetic analysis based on combined mitochondrial gene datasets showed that the five *Bipolaris* species formed well-supported topologies. This study is the first report on the mitogenomes of *B. maydis* and *B. zeicola*, as well as the first comparison of mitogenomes among *Bipolaris* species. The findings of this study will further advance investigations into the population genetics, evolution, and genomics of *Bipolaris* species.

**Supplementary Information:**

The online version contains supplementary material available at 10.1186/s43008-024-00149-6.

## Introduction

The genus *Bipolaris* includes many important plant pathogens with a wide host range and worldwide distribution (Manamgoda et al. 2014; Manamgoda et al. 2012; Manamgoda et al. 2011). These species mainly affect high value field crops in the *Poaceae* family, causing different typical symptoms such as leaf spot, leaf blight, seedling blight and root rot (Manamgoda et al. 2011; Ellis et al. 1971; Berbee et al. 1999). In the past, devastating diseases caused by five *Bipolaris* species (*Bipolaris maydis*, *B. zeicola*, *B. oryzae*, *B. sorokiniana*, and *B. cookei*) on staple crops such as maize, rice and wheat have led to huge economic losses and food crises. For example, southern corn leaf blight (SCLB) caused by *B. maydis* caused great damage in the Midwestern United States in the late 1970s (Rossman et al. 2013). Northern corn leaf spot (NCLS), caused by *B. zeicola*, was a widespread leaf disease of maize and grass crops in many parts of the world, and had become an important factor affecting world maize production (Liu et al. 2015). The Bengal famine in India (1943-1944) was the result of a rice disease caused by *B. oryzae* (Ou et al. 1985; Scheffer et al. 1997). In 1990, *B. sorokiniana* was declared the most important foliar pathogen of wheat in warm regions of the world (Duveiller et al. 1994). In addition, *B. cookei* had been associated with severe losses in *Sorghum* production (Zummo et al. 1987). Therefore, the accurate identification of *Bipolaris* species and the study of control methods is one of the recent hotspots of interest for plant pathologists.

The genus *Bipolaris* belongs to *Ascomycota*, *Dothideomycetes*, *Pleosporales*, *Pleosporaceae* (Manamgoda et al. 2014; Bhunjun et al. 2020). *Bipolaris* species were previously described in *Helminthosporium*, which were later separated into several genera including *Bipolaris*, *Curvularia*, *Drechslera*, *Exserohilum*, *Johnalcornia* and *Porocercospora* (Bhunjun et al. 2020; Sivanesan 1987). Its sexual morph, the genus *Cochliobolus*, was also the older name, however, the scientific name *Bipolaris* was generally recommended for use according to the International Code of Nomenclature for algae, fungi, and plants, and plant pathologists also tended to prefer it (Manamgoda et al. 2011; Rossman et al. 2013). Taxonomy of *Bipolaris* species and their neighbouring genera has traditionally relied on morphological features. However, since most *Bipolaris* species can’t produce sexual morph under natural and laboratory conditions, and genera *Bipolaris* and *Curvularia* are morphologically similar, therefore the identification of *Bipolaris* species using morphological and biological species concepts is not always accurate (Manamgoda et al. 2014; Manamgoda et al. 2011). In addition, the problem of using only ITS BLAST searches leading to misidentification of species and thus mislocalization at the species or genus level has hindered accurate molecular identification of species in the *Bipolaris* genus (Bhunjun et al. 2020). Subsequently, Bhunjun et al. suggested that GAPDH-based phylogenetic analyses could be performed using correct taxon sampling to obtain accurate results (Bhunjun et al. 2020). All in all, the accurate identification and classification of *Bipolaris* species is of great importance for the discovery, control and utilization of *Bipolaris* species.

Mitochondria are double-membrane organelles found in eukaryotes whose main role is to provide energy for cellular life activities (Ernster et al. 1981; McBride et al. 2006; Murphy et al. 2009). Mitochondria contain their own genome (mitogenome), known as the “second genome”, and are thought to have been obtained from alpha-proteobacteria through endosymbiosis (Gray et al. 2001). Because of the maternal inheritance, rapid evolution, low recombination rates, and many available molecular markers, the mitogenome is an effective tool for inferring species phylogenetic relationships, population genetics, and comparative genomics studies (Abuduaini et al. 2021; Basse et al. 2010; Burger et al. 2003; Bullerwell et al. 2005; Li et al. 2022; Li et al. 2019). In recent years, the development of high-throughput sequencing technology and related bioinformatics tools have improved the study of mitogenomes (Song et al. 2020; Mardanov et al. 2014). Nonetheless, the number of fungal mitogenomes available in databases is much less than that of animals (https://www.ncbi.nlm.nih.gov/genome/browse#!/organelles/). Mitogenomes of different fungal species have been reported to have significant differences in genome size, composition, gene arrangement, repeat sequence content, and number of introns and open reading frames (ORFs), even among closely related species (Fonseca et al. 2021; Ren et al. 2021; Wu et al. 2020a; Li et al. 2022). Despite these differences, most fungal mitogenomes contain a conserved set of protein-coding genes (PCGs), including *atp6*, *atp8*, *atp9*, *cob*, *cox1*, *cox2*, *cox3*, *nad1*, *nad2*, *nad3*, *nad4*, *nad4L*, *nad5*, *nad6*, and *rps3* (Ma et al. 2022). These genes play critical roles in maintaining cellular homeostasis and cellular energy supply (Chatre et al. 2014; Osiewacz et al. 2002). In addition, there are two types of introns in the fungal mitogenomes, group I and group II, with group I being the most common and usually encoding two types of homing endonuclease genes (HEGs) containing either LAGLIDADG or GIY-YIG motifs (Brankovics et al. 2017; Belfort et al. 2014). A growing body of evidence suggests that the length and number of introns and repetitive fragments, as well as horizontal transfer of genes, contribute to the large variation in fungal mitogenome size (Kanzi et al. 2016; Himmelstrand et al. 2014). To date, only three *Bipolaris* species (*B. sorokiniana*, *B. cookei*, and *B. oryzae*) mitogenomes have been reported. Among them, previous researchers conducted detailed analysis on the mitogenomes of *B. sorokiniana* and *B. cookei*, while *B. oryzae* only reported its mitogenome sequence. These results contribute to our initial understanding of the mitogenome characteristics of the genus *Bipolaris* (Zaccaron et al. 2017; Zhang et al. 2023; Deng et al. 2019). However, there are still many critical *Bipolaris* species whose mitogenomes are unknown, and comparative analyses between mitogenomes of *Bipolaris* species are also lacking.

In the present study, the mitogenomes of three *Bipolaris* phytopathogens (*B. maydis*, *B. zeicola* and *B. oryzae*) were sequenced, assembled, annotated, and compared with the mitogenomes of the other two reported *Bipolaris* species (*B. sorokiniana* and *B. cookei*) (Zaccaron et al. 2017; Zhang et al. 2023). The aims of this study were (1) to reveal the characterizations of the *B. maydis* and *B. zeicola* mitogenomes; (2) to compare the mitogenome structure, contents, and gene orders of five *Bipolaris* species to assess variation and conservation among *Bipolaris* species; and (3) to understand the phylogenetic positions of the five existing *Bipolaris* species in the *Ascomycota* based on the combined mitochondrial gene set. This study not only enriches the mitogenome data of the *Bipolaris* but also provides information for understanding the genetic evolution of the *Bipolaris* species.

## Materials and methods

### Sample collection and DNA extraction

With the method of tissue isolation (Lin et al. 2011), the strains *B. maydis* and *B. zeicola* were isolated from maize leaves with diseased spots in Henan Province, China. Additionally, strains of *B. oryzae* were isolated from diseased rice leaves in the same province. The hyphal tips were first transferred to PDA plates for purification, and then purified mycelia were kept in 30 % aqueous glycerol and eventually stored at -80 ° C in the fungal collection of Henan Agricultural University. Species identification was based on morphological characters and sequence analysis of ITS, *tef1* and GAPDH genes. These sequences were deposited in GenBank under the accession numbers listed in Additional file 1: Table S1. Total genomic DNA (gDNA) extraction of the three specimens collected by us was performed using cetyl trimethyl ammonium bromide (CTAB) method (Manamgoda et al. 2012). To ensure that each DNA sample was of adequate quality for PCR, UV spectrophotometry (Nanodrop 2000, Thermo, Wilmington, DE) was used to determine DNA concentration and purity.

### Mitogenome sequencing and assembly

High-quality gDNA was sent to Novogene Co., Ltd. for genome sequencing. Short insert libraries (350 bp) were constructed using NEBNext Ultra DNA Library Prep Kit for Illumina (NEB, USA). Whole genome sequencing was performed on the Illumina Hiseq X Ten platform (Novogene, Tianjin, China), generating 150 bp paired-end reads for each sample. Adapters and low-quality reads were trimmed using fastp v0.13.1 (Chen et al. 2018). This was followed by de-duplication and error correction using FastUniq v1.1 and Musket v1.1 (Xu et al. 2012; Liu et al. 2013). The cleaned paired-end reads were de novo assembled using SPAdes v3.14.1 software with k-mers of 21, 33, 55, 77, 99 and 127 (Bankevich et al. 2012). Mitochondria-related contigs in each sample were then identified and pooled by BLASTn search using *B. cookei* mitogenome as a reference (Zaccaron et al. 2017). Finally, three circularly assembled mitogenomes of the genus *Bipolaris* were further obtained using MITObim v1.9 (Hahn et al. 2013). In addition, three assembled mitogenome sequences were verified by NOVOPlasty (Dierckxsens et al. 2017).

### Annotation of mitogenomes

Three obtained complete mitogenomes were annotated according to the method described by Wu et al (Wu et al. 2020b). First, we initially annotated PCGs, open reading frames (ORFs), rRNAs, tRNAs, and introns of the three *Bipolaris* mitogenomes using MFannot and MITOS, both based on genetic code 4 (Valach et al. 2014; Bernt et al. 2013). The PCGs and ORFs were further modified or predicted using the ORF Finder (https://www.ncbi.nlm.nih.gov/orffinder) and annotated by BLASTP searches against the NCBI non-redundant protein sequence database (Bleasby et al. 1990). Next, we used the *B. cookei* mitogenome as a reference to detect intron and exon boundaries using exonerate v2.2 (Zaccaron et al. 2017; Slater et al. 2005). The previously further annotated tRNA genes were further verified by tRNAscan-SE v2.00 (Lowe et al. 2016). Finally, graphical maps of the three *Bipolaris* mitogenomes were drawn using OGDraw v1.3.1 (Greiner et al. 2019). In addition, the complete mitogenomes of 13 additional *Pleosporales* species were downloaded from the NCBI database and mitogenomes of *B. sorokiniana* and *B. cookei* were reannotated for further comparative analysis.

### Analysis of sequences and repetitive elements

The frequency of codon use, preference and base composition of mitogenomes of five *Bipolaris* species were analyzed using the sequence manipulation suite, based on genetic code 4 (http://www.bioinformatics.org/sms2/codon_usage.html). DnaSP v5.10 software was used to calculate synonyms ( *Ks* ) and non-synonymous substitution rates ( *Ka* ) for 12 core PCGs (*atp6*、*cob*、*cox1*、*cox2*、*cox3*、*nad1*、*nad2*、*nad3*、*nad4*、*nad4L*、*nad5*、*nad6*) in mitogenomes of all 16 acquired *Pleosporales* (Librado et al. 2009. Based on the Kimura-2 parameter (K2P) substitution model, the overall mean genetic distances between each pair of 12 core PCGs were calculated with MEGA v6.06 (Caspermeyer 2016). Mauve v2.4.0 software was used to perform mitogenome collinearity analysis of five *Bipolaris* species (Darling et al. 2004). To detect interspersed repeats or intragenomic duplications of large fragments in the mitogenomes of the five *Bipolaris* species, we conducted BLASTn searches against each mitogenome against itself, with an E-value of < 1e-10 (Chen et al. 2015). Tandem Repeats Finder was used to detect tandem repeats within the five *Bipolaris* mitogenomes (Benson 1999).

### Comparative mitogenomic and intron analysis

Comparative mitogenome analyses were performed to assess the variations and conservations of the genus *Bipolaris* with other *Pleosporales* species in terms of genome size, base composition, GC content, number of genes, number of introns, gene arrangement, and gene content. The *cox1* gene introns of the mitogenome could be classified into different position classes (Pcls) (Férandon et al. 2010). The same Pcls from different species usually had high sequence similarity and contain homologous intronic ORFs. To detect intron insertion sites, the *cox1* gene of 16 *Pleosporales* species was compared using Clustal W using the *cox1* gene of *B. cookei* as a reference (Zaccaron et al. 2017; Thompson et al. 1994). Pcls were named according to their insertion sites in the corresponding reference sequences.

### Mitochondrial phylogenetic analysis

To investigate the phylogenetic positions of these five *Bipolaris* species, we constructed a phylogenetic tree of 96 ascomycetes using the concatenated mitochondrial gene set (including 14 core PCGs and one *rps3* gene). Both *Taphrina deforman* and *T. wiesneri* of the *Taphrinomycetes* were appointed as outgroups (Tsai et al. 2014). Individual mitochondrial genes were first aligned with Clustal W using MEGA v6.06 software, and then concatenated into a combined mitochondrial gene set using FASconCAT v1.0 (Caspermeyer 2016; Kück et al. 2010). The Partition homogeneity test was used to detect potential phylogenetic conflicts between different mitochondrial gene. Partition Finder v2.1.1 was used to determine the best evolutionary model for combined alignment (Lanfear et al. 2017). Phylogenetic trees were constructed using Bayesian inference (BI) and Maximum-Likelihood (ML). The BI analysis was performed using MrBayes v.3.1.2 with GTR substitution model and gamma-distributed rate variation for the site-to-site variance ratio, where the portion was a proportion of invariant sites and the rest of the parameters were default values, and the mcmc method was used to calculate 3,000,000 generations, sampling once every 100 generations, discarding 25 % of the aged samples from the obtained samples, and the remaining trees were used to calculate Bayesian posterior probability (BPP) values in the 50 % majority-rule consensus trees (Ronquist et al. 2003). The ML analysis was performed in IQtree v.1.6.8. Bootstrap values (BS) were evaluated using an ultrafast bootstrap approach with 1000 replicates under the GTR+G substitution model (Nguyen et al. 2015). Phylogenetic trees were visualized and adjusted in FigTree v. 1.4.2 (http://tree.bio.ed.ac.uk/software/figtree/).

### Data availability

The complete mitogenomes of *B. maydis*, *B. zeicola* and *B. oryzae* were deposited in the GenBank database under the accession numbers OR695074, OR695075 and OR695076, respectively.

## Results

### Features, genetic compositions and PCGs of five *Bipolaris* mitogenomes

**Fig. 1:**
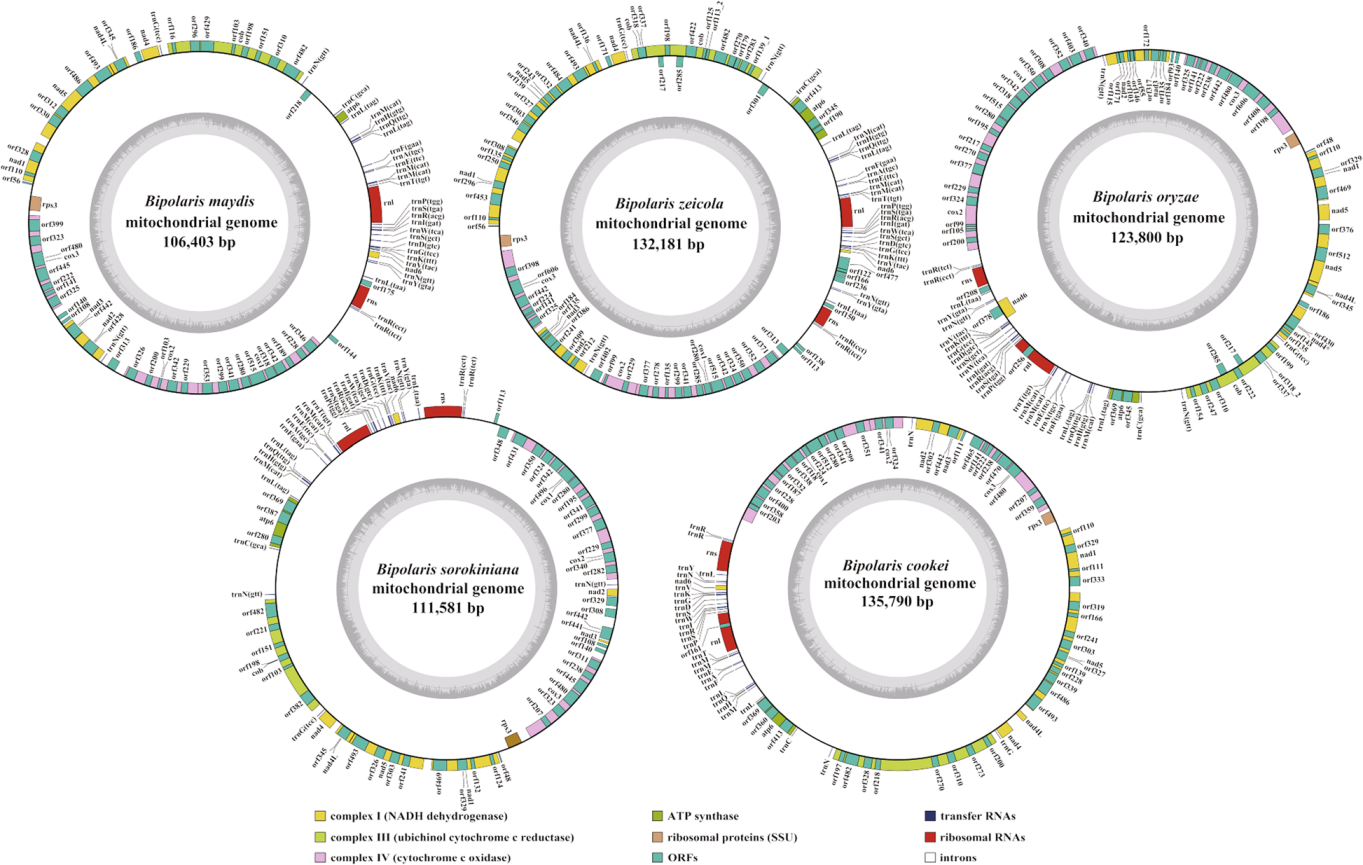
Circular maps of the five *Bipolaris* mitogenomes. Genes are represented by different colored blocks. Colored blocks outside each ring indicate that the genes are on the direct strand, while colored blocks within the ring indicates that the genes are located on the reverse strand. Genes on the direct strand are transcribed in a counterclockwise direction, while genes on the reverse strand are transcribed in a clockwise direction. The inner grayscale bar graph shows the GC content of the mitochondrial sequences. The circle inside the GC content graph marks the 50% threshold

In this study, we newly assembled the complete mitogenomes of three *Bipolaris* species, *B. maydis*, *B. zeicola*, and *B. oryzae*, with sizes of 106,403 bp, 132,181 bp, and 123,800 bp, respectively, of which *B. maydis* and *B. zeicola* mitogenomes were assembled for the first time. In addition, mitogenomes of *B. sorokiniana* and *B. cookei* were downloaded from public databases with sizes of 111,581bp and 135,790bp, respectively, and compared and analyzed with mitogenomes of the three newly assembled *Bipolaris* species (Fig. 1). The GC content of strains *B. maydis*, *B. zeicola*, *B. oryzae*, *B. sorokiniana* and *B. cookei* were very close to each other with 30.13%, 30.22%, 30.30%, 30.41% and 30.00%, respectively (Additional file 1: Table S2). Except the *B. oryzae* mitogenome, which both AT skews and GC skews were positive, the other four *Bipolaris* species were negative for AT skews and positive for GC skews. As for the genetic composition of the genome, the five *Bipolaris* species shared the same trend, i.e., the highest content of intronic regions, the second and third highest content of intergenic and protein coding regions, and the smallest content of ncRNA genes (tRNAs and rRNAs) (Additional file 2: Fig. S1). In addition, a total of 15 to 21 free-stranding PCGs were detected in each of the five *Bipolaris* mitogenomes, with *B. maydis* having the highest number and *B. cookei* the lowest (Additional file 1: Table S2 and S3). All mitogenomes contained 12 typical core PCGs (*atp6*, *cob*, *cox1*, *cox2, cox3, nad1, nad2, nad3, nad4, nad4L, nad5, and nad6*) and one *rps3* gene. Interestingly, in these core PCGs, *cox1* was seamlessly adjacent to the *cox2* gene, resembling a fused gene. Meanwhile, we found that non-conserved PCGs in the *Bipolaris* mitogenomes partially encode homing endonucleases (HEs) and the other encode proteins with unknown function.

We also detected the number of introns ranging from 34 to 45 in the five mitogenomes, with *B. oryzae* containing the highest number of introns and *B. sorokiniana* the lowest (Additional file 1: Table S2 and S3). Meanwhile, 39 intronic ORFs were found in the mitogenome of *B. maydis*, 63 in *B. zeicola*, 59 in *B. oryzae*, 40 in *B. sorokiniana* and 50 in *B. cookei*. Most of these intronic ORFs encoded HEs with LAGLIDADG endonuclease motifs, and a few encoded HEs with GIY-YIG motifs and proteins with unknown function.

### RNA genes in the *Bipolaris* mitogenomes

The five *Bipolaris* mitogenomes all contained two rRNA genes, small-subunit ribosomal RNA (*rns*) and large-subunit ribosomal RNA (*rnl*) (Additional file 1: Table S3). With the exception of one intron in the *rnl* gene in each mitogenome of *B. oryzae* and *B. cookei*, none of the other *rnl* and *rns* genes in the five *Bipolaris* species contained introns. In addition, the longest *rns* (3,659 bp) and *rnl* (4,868 bp) were found in mitogenomes of *B. cookei* and *B. oryzae*, respectively (Additional file 1: Table S3). The average lengths of the *rns* and *rnl* genes were 2,440 bp and 4,003 bp, respectively.

**Fig. 2:**
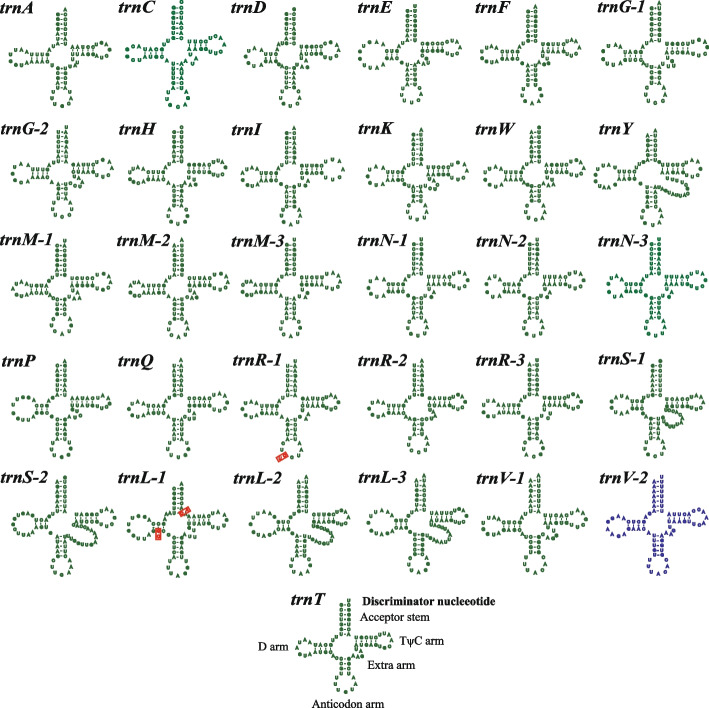
Putative secondary structures of tRNA genes identified in the mitogenomes of five *Bipolaris* species. The 29 tRNAs in green or red fonts represent tRNAs shared by the five *Bipolaris* species, while the tRNA in blue font represent tRNA only in *Bipolaris maydis*, *B. zeicola*, and *B. sorokiniana*. Residues conserved across the five mitogenomes are shown in green, while variable sites are shown in red

We detected 31 tRNA genes in the mitogenomes of *B. maydis*, *B. zeicola*, and *B. sorokiniana*, and 30 tRNA genes in the mitogenomes of *B. oryzae* and *B. cookei* (Additional file 1: Table S2). These tRNA genes encoded 20 canonical amino acids and could be folded into the classical cloverleaf structure. The size of individual tRNA genes ranged from 71 bp to 85 bp, mainly due to variation in the size of the extra arms (Additional file 1: Table S3). At the same time, three (*trnM*, *trnN*, *trnR* and *trnL*) and two (*trnG* and *trnS*) tRNA genes were duplicated in all five mitogenomes. A copy of the (*trnA*, *trnC*, *trnD*, *trnE*, *trnF*, *trnH*, *trnI*, *trnK*, *trnW*, *trnY*, *trnP*, *trnQ* and *trnT*) tRNA gene was present in the five mitogenomes (Fig. 2). Overall, the mitogenomes of *B. maydis*, *B. zeicola*, and *B. sorokiniana* contained an additional tRNA gene, *trnV-2*, compared to the mitogenomes of *B. cookei* and *B. oryzae*. Of the 30 tRNA genes shared by the five mitogenomes, there are three variable sites, one of which is located in the *trnR-1* anticodon arm, and two in the *trnL-1* D-arm and acceptor stem, respectively.

### Codon usage analysis

**Fig. 3:**
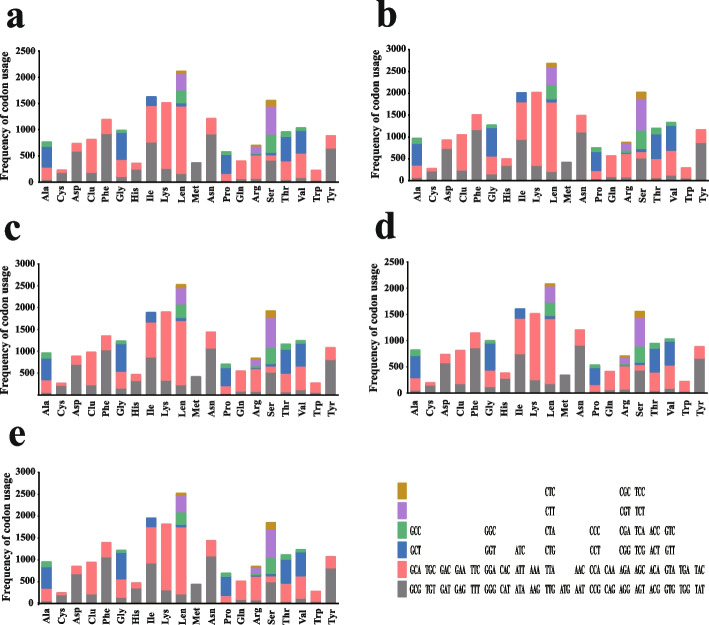
Codon usage in the mitogenomes of five *Bipolaris* species. Frequency of codon usage is plotted on the y-axis. **a**: *Bipolaris maydis*, **b**: *B. zeicola*, **c**: *B. oryzae*, **d**: *B. sorokiniana*, **e**: *B. cookie*

ATG was the only start codon used in the 13 core PCGs of the five *Bipolaris* mitogenomes (Additional file 1: Table S4). TAA (66.15%) was the most commonly used stop codon in the core PCGs of five *Bipolaris* mitogenomes, followed by TAG (33.85%). The stop codons varied among the five *Bipolaris* species. i.e., the *nad4* genes of *B. maydis*, *B. zeicola*, *B. oryzae* and *B. cookei* had TAA as a stop codon, while *B. sorokiniana* used TAG as a stop codon. The *nad5* gene of *B. cookei* had TAG as a stop codon, and the *nad5* gene of the other four species had TAA as a stop codon. Codon usage analysis showed that the codons most frequently used in the five *Bipolaris* mitogenomes were TTA (for leucine; Leu), AAA (for lysine; Lys), AAT (for asparagine; Asn), TTT (for phenylalanine; Phe), ATA (for isoleucine; Ile), ATT (for isoleucine; Ile), and TAT (for tyrosine; Tyr) (Fig. 3 and Additional file 1: Table S5). The frequent use of A and T in codons was responsible for the high AT content in the *Bipolaris* mitogenomes (69.79% on average).

### Overlapping nucleotides, intergenic regions and repeat elements analysis

Two pairs of overlapping nucleotides were detected in the *B. cookei* mitogenome between adjacent genes *orf333* and *nad1* ( -1,146 bp), and *cox3* and *orf465* ( -1,193 bp), whereas only one pair of overlapping nucleotides existed in the other four *Bipolaris* mitogenomes between adjacent genes *nad4L* and *nad5* ( -1 bp) (Additional file 1: Table S3).

Intergenic region sequences ranging from 22,267 bp to 27,753 bp were detected in the five *Bipolaris* mitogenomes, with occupations ranging from 17.99% to 24.56%. The longest intergenic segments in the *B. maydis*, *B. zeicola*, *B. oryzae*, *B. sorokiniana* and *B. cookei* mitogenomes were located between *trnR* and *orf144* (3,274bp), *trnC* and *orf301* (3,056bp), *rnpB* and *rnC* (3,309bp), *rnpB* and *rnC* (3,317bp), and *trnC* and *trnN* (4,969bp), respectively.

We conducted BlastN searches of the five *Bipolaris* mitogenomes against themselves, and identified 157, 228, 214, 158, and 274 repetitive elements in the mitogenomes of *B. maydis*, *B. zeicola*, *B. oryzae*, *B. sorokiniana* and *B. cookei*, respectively (Additional file 1: Table S6). The length of these repeat elements ranged from 38 to 1,054 bp, with pairwise nucleotide similarities ranging from 64.24% to 100%. The largest repeats (1,054bp) were found in the intronic regions of the *nad5* and *cox3* genes of *B. maydis*, *B. oryzae*, *B. sorokiniana* and *B. cookei* mitogenomes. While the largest repeats in the mitogenome of *B. zeicola* were 695 bp which were located in the intronic regions of *nad5* and *nad6*, respectively. Repetitive sequences accounted for 20.31%, 22.29%, 24.67%, 19.21%, and 23.21% of the *B. maydis*, *B. zeicola*, *B. oryzae*, *B. sorokiniana* and *B. cookei* mitogenomes, respectively. In addition, 44, 40, 40, 36 and 34 tandem repeats were identified in *B. maydis*, *B. oryzae*, *B. sorokiniana* and *B. cookei* mitogenomes, respectively (Additional file 1: Table S7). The longest tandem repeat sequence (249 bp) was detected in the mitogenomes of *B. maydis* and *B. zeicola* which were located in the intergenic region between *trnL* and *trnY*. Tandem repeat sequences accounted for 3.24%, 2.26%, 2.42%, 2.45%, and 2.06% of the *B. maydis*, *B. zeicola*, *B. oryzae*, *B. sorokiniana* and *B. cookei* mitogenomes, respectively.

### Intron dynamics of *cox1* genes

A total of 379 introns were detected in 16 Pleosporales mitogenome PCGs, most of which belonged to the group I (Additional file 1: Table S2 and S3). These introns were unevenly distributed in the host genes, with a clear preference for particular host genes. i.e., the *cox1* gene tended to be introns-rich, containing 118 introns, accounting for 31.13% of the total introns in the 16 *Pleosporales* mitogenomes. Thus, intron dynamics in the *cox1* gene could significantly affect the organization and size of *Pleosporales* mitogenomes.

**Fig. 4:**
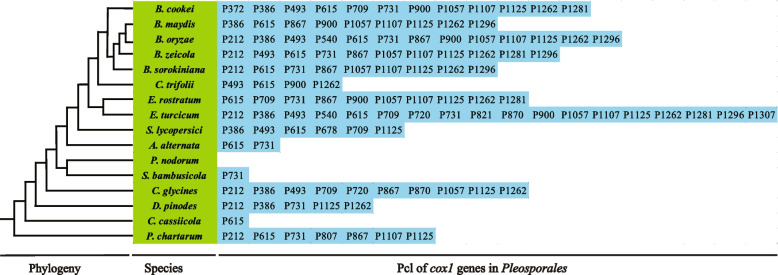
Position class (Pcl) information of *cox1* genes in the 16 species. Introns in *cox1* genes of 16 published mitogenomes were classified into different position classes (Pcls) using the *cox1* gene of *Bipolaris cookei* as the reference. Each Pcl was constituted by introns inserted at the same position of corresponding *cox1* gene and named according to its insertion site in the aligned corresponding reference sequence (nt). Phylogenetic positions of the 16 species were established using the Bayesian inference (BI) method and Maximum-Likelihood (ML) method based on combined mitochondrial data sets

Following the method described by Férandon et al. and using *cox1* gene of the *B. cookei* as a reference (Zaccaron et al. 2017; Férandon et al. 2010), we classified the introns in *cox1* genes of 16 *Pleosporales* mitogenome into different Pcls. The 118 introns in the *cox1* genes of 16 mitogenomes were classified into 22 Pcl types, indicating the rich diversity of intron types in *Pleosporales* (Additional file 2: Fig. S2). The number of introns varied considerably between species, with the highest number of Pcls (18) in the *cox1* gene of *Exserohilum turcicum* and the lowest number of Pcls (0) in *Phaeosphaeria nodorum* (Fig. 4). It has been shown that intron loss/gain events may have occurred in the course of intron evolution in different *Pleosporales* species. The most widely distributed intron was P615, which was distributed in 12 of the 16 *Pleosporales* species. Introns P1125 and P1262 were the second and third most common introns, found in 11 and 10 of the 16 *Pleosporales* mitogenomes, respectively. Some Pcls were detected in only one of the 16 *Pleosporales* species, such as P372, P678, P807, P821 and P1307. These results indicated that *Pleosporales*’ ancestors lost or gained introns to a large extent during evolution. In addition, 54 Pcls were detected in the *cox1* genes of five *Bipolaris* species, accounting for 45.76% of the total introns in the *cox1* gene of 16 *Pleosporales* species. This suggests that the *cox1* genes of the five *Bipolaris* species contains a higher number of Pcls, with *B. oryzae* having the highest number of Pcls (13) and *B. maydis* and *B. sorokiniana* having the lowest number of Pcls (9). Meanwhile, Pcls P615, P1057, P1107, and P1262 were found to be distributed in five *Bipolaris* species, and P731, P867, P1125, and P1296 were detected in four of the five *Bipolaris* species, suggesting that these Pcls might be prevalent in *Bipolaris* species.

### Genetic distances, evolutionary rates and variations of core PCGs

**Fig. 5:**
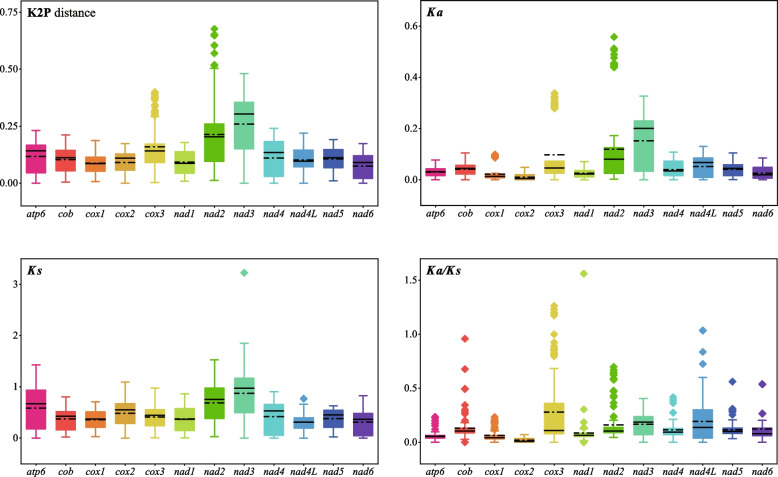
Genetic analysis of 12 core protein coding genes (excluding *rps3* gene) in 16 *Pleosporales* species. The black straight and dotted lines indicate the magnitude of the median and mean values, respectively. K2P, the Kimura-2-parameter distance; *Ka*, the number of nonsynonymous substitutions per nonsynonymous site; *Ks*, the number of synonymous substitutions per synonymous site

Since the mitogenomes of some *Pleosporales* species do not contain the *rps3* gene, here only 12 core PCGs were used to calculate the K2P genetic distances and substitution rates between each pair of the 16 *Pleosporales* species (Fig. 5). Of the 12 PCGs detected, *nad3* had the largest average K2P genetic distance between the 16 *Pleosporales* species (average values of 0.2587), followed by *nad2* and *cox3* (average values of 0.214 and 0.1599), suggesting that these genes diverged greatly in evolution. In contrast, the *nad6* and *cox1* genes had the smallest average K2P genetic distances (average values of 0.0751 and 0.0874), indicating that these genes are highly conserved. Among the 12 core PCGs detected, the *nad3* gene showed the largest *Ka* (average values of 0.1525), while the *cox2* gene had the smallest *Ka* value (average values of 0.0103). The *nad3* gene had the highest *Ks* (mean value 0.8754), while the *nad4L* gene had the lowest *Ks* value (mean value 0.3063) among the 16 *Pleosporales* species. The *Ka/Ks* values for all the 12 core PCGs were well below 1, indicating that these genes underwent purifying selection during evolution.

**Fig. 6:**
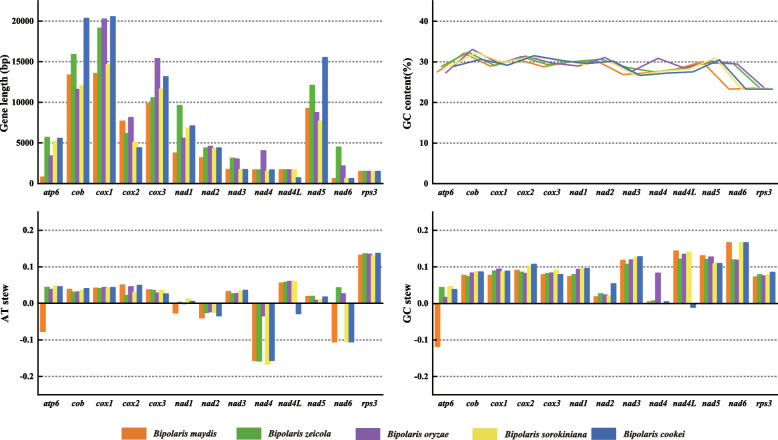
Sequence information of 13 core protein coding genes in the five *Bipolaris* species

At the same time, we also made an in-depth comparison of the similarities and differences in the 13 core PCGs (including *rps3* gene) between five *Bipolaris* species. Of the 13 core PCGs, except for *rps3*, which had identical gene lengths in the five *Bipolaris* species, the remaining 12 PCGs had sequence length variations, with the *cob* having the largest length variation of 8,744 bp (Fig. 6). Among the 13 core PCGs detected, *cob* had the highest average GC content of 32.09%, followed by *cox2* with 31.23%. The *rps3* gene had the lowest GC content with an average of 23.52%. In addition, there were differences in GC content of the same PCGs in the five mitogenomes. i.e., *cob* contained the highest GC content in *B. maydis*, *B. zeicola*, *B. oryzae*, and *B. sorokiniana* (32.12%, 32.36%, 33.08%, and 32.26%), while *cox2* contained the highest GC content in *B. cookei* (31.53%). The *rps3* had the lowest GC content in *B. zeicola* and *B. oryzae* (23.56% and 23.62%), while *nad6* was the gene with the lowest GC content in *B. maydis*, *B. sorokiniana* and *B. cookei* (23.28%, 23.28% and 23.28%). These differences indicate that the mitochondrial core PCGs of the *Bipolaris* frequently exhibit base variations.

The AT skews of *cob*, *cox1*, *cox2*, *cox3*, *nad3*, *nad5*, and *rps3* in the five mitogenomes were positive, and the AT skews of *nad2* and *nad4* were negative (Fig. 6). The difference was that *atp6* and *nad1* showed negative AT skew in *B. maydis* and positive AT skew in the other four *Bipolaris* species. The *nad4L* showed negative AT skew in *B. cookei* and positive AT skew in the other four *Bipolaris* species. The *nad6* showed positive AT skew in *B. zeicola* and *B. oryzae* and negative AT skew in the other three *Bipolaris* species. AT skew in core PCGs varied among species within the genus, indicating frequent A/T mutations in core PCGs. Most PCGs were positive for GC skew, except for the *atp6* gene in *B. maydis* and the *nad4L* gene in *B. cookei*, which were negative, suggesting that most core PCGs tended to evolve towards G-rich rather than C-rich in the leading strand of the core PCGs (Fig. 6).

### Gene arrangement

**Fig. 7:**
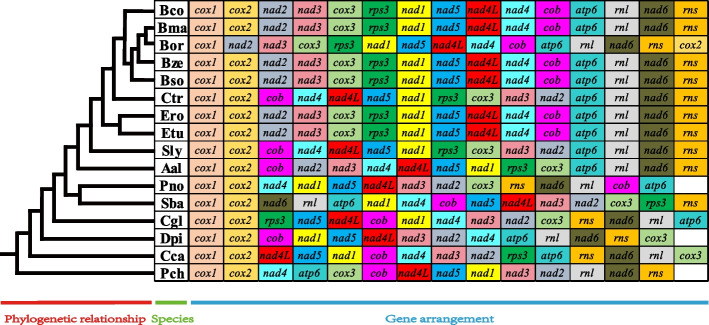
Mitochondrial gene arrangement analyses of the 16 *Pleosporales* species. The gene sequence begins with the *cox1* gene and contains 13 core protein coding genes (PCGs) and two rRNA genes. The same gene were represented by same color blocks. Phylogenetic positions of the 16 species were established using the Bayesian inference (BI) method and Maximum-Likelihood (ML) method based on combined mitochondrial data sets

We further compared mitochondrial gene arrangements in 16 *Pleosporales* species, including 13 core PCGs and 2 rRNA genes. The results showed that large-scale mitochondrial gene rearrangements were detected between species of different genera (Fig. 7). Four uninterrupted gene pairs, *cox1* and *cox2*, *nad2* and *nad3*, *atp6* and *rnl*, *nad4L* and *nad5*, were also found in the mitochondrial gene arrangement of 16 *Pleosporales* species. Meanwhile, the mitochondrial gene arrangement of *B. maydis*, *B. zeicola*, *B. sorokiniana* and *B. cookei* among the five *Bipolaris* species was identical, but within the mitochondrial genes of *B. oryzae*, inversion of *cox1* and *cox2* gene pairs was observed. In addition, *E. turcicum* and *E. rostratum* share the same gene arrangement as *B. maydis*, *B. zeicola*, *B. sorokiniana* and *B. cookei*, suggesting that *Exserohilum* is phylogenetically closely related to *Bipolaris* (Fig. 7). In contrast to the genus *Bipolaris*, a vastly different set of gene arrangements was found in *Curvularia trifolii* of the genus *Curvularia* (Fig. 7). This variation was due to the inversion of gene fragments between *cob* to *nad2*.

A total of six locally collinear blocks (A to F) were detected in five *Bipolaris* mitogenomes based on the analysis in Mauve (Additional file 2: Fig. S3). Except for the rearrangement of homologous regions C and E in the mitogenome of *B. oryzae*, the other homologous regions were of similar size and in the same order among the five *Bipolaris* species. In summary, the gene arrangement and collinearity in the mitogenomes of the five *Bipolaris* species were almost highly conserved within the genus.

### Comparative mitogenomic analysis

Of the 16 *Pleosporales* species detected, there was a large variation in the mitogenomes, with *E. turcicum* having the largest mitogenome (264,948 bp), and *Shiraia bambusicola* having the smallest (39,030 bp), suggesting that *Pleosporales* mitogenomes have undergone large expansion/contraction during evolution (Additional file 1: Table S2). The sizes of the five *Bipolaris* mitogenomes were located in the 2nd to 6th positions, which was only smaller than that of *E. turcicum*, but all were higher than the average size of the 16 *Pleosporales* (92,338.5 bp). Meanwhile, the GC content of the 16 mitogenomes ranged from 25.19% to 30.41%, with an average of 29.30% (Additional file 1: Table S2). The five *Bipolaris* mitogenomes contained the highest GC content of the 16 mitogenomes, ranging from 30.00% to 30.41%, which was significantly higher than the average of the 16 mitogenomes. Eight and fourteen of 16 *Pleosporales* mitogenomes had positive AT skews and GC skews, respectively. The number of PCGs in the 16 mitogenomes ranged from 15 to 49, with *E. turcicum* having the highest number of PCGs and *Corynespora cassiicola* and *B. cookei* having the lowest. All 16 mitogenomes contained two rRNA genes. In addition, 25 to 32 tRNA genes were detected in 16 *Pleosporales* species.

### Phylogenetic analysis

**Fig. 8:**
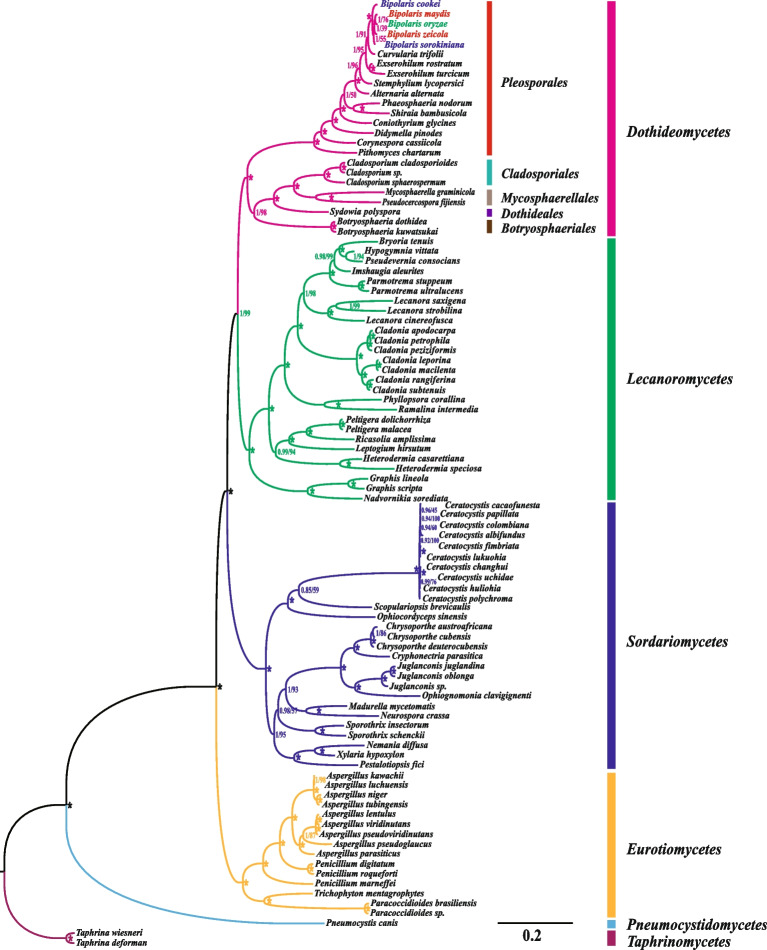
Molecular phylogeny of 94 *Ascomycota* species based on Bayesian inference (BI) and Maximum likelihood (ML) analysis of 15 protein coding genes. Support values are Bayesian posterior probabilities (BPP) and bootstrap values (BS) placed before and after the slash, respectively. Asterisks indicate BPP and BS values of 1.00 and 100, respectively. Species and NCBI registry numbers of mitogenomes used for phylogenetic analyses can be provided in Additional file 1: Table S8

Phylogenetic analyses using BI and ML methods based on the 15 concatenated mitochondrial conserved PCG genes (including *atp6*, *atp8*, *atp9*, *cob*, *cox1*, *cox2*, *cox3*, *nad1*, *nad2*, *nad3*, *nad4*, *nad4L*, *nad5*, *nad6*, and *rps3* gene) obtained an identical and well-supported phylogenetic tree (Fig. 8). All major clades within the phylogenetic tree were well supported (BPP ≥ 0.85; BS ≥ 50). Both *T. deforman* and *T. wiesneri* from *Taphrinomycetes* were designated as outgroups, while the other 96 *Ascomycota* species were divided into five major clades, corresponding to *Dothideomycetes*, *Sordariomycetes*, *Lecanoromycetes*, *Eurotiomycetes* and *Pneumocystidomycetes* (Additional file 1: Table S8). Within the *Dothideomycetes*, members of *Pleosporales* were also well separated from members of neighbouring *Cladosporiales*, *Mycosphaerellales*, *Dothideales* and *Botryosphaeriales*. Of the 16 *Pleosporales* species, the different members were well separated and had good BS support among them as well as BPP values of 1 for all clades. Among them, members of *Bipolaris*, *Curvularia*, and *Exserohilum* were close in the evolutionary tree, suggesting they had a close relationship. Meanwhile, members of the same genus clustered together, suggesting that the five *Bipolaris* species were sister species of *Bipolaris*. In conclusion, phylogenetic analyses suggested that the mitogenomes had the potential to help taxonomists distinguish between members of *Bipolaris* and its neighbouring genera.

## Discussion

### Mitogenome size variation in *Bipolaris* species

In this study, we sequenced and assembled the mitogenomes of *B. maydis*, *B. zeicola*, and *B. oryzae* from the genus *Bipolaris*, and compared and analyzed them with those of *B. sorokiniana* and *B. cookei*, as well as eleven other *Pleosporales* species, as previously reported (Zhang et al. 2023; Deng et al. 2019; Ma et al. 2022). Mitogenomes of *Pleosporales* species varied greatly in size, with the largest mitogenome being 6.79 times larger than the smallest mitogenome (Ma et al. 2022; Shen et al. 2015). Large genomic size variability was also detected among mitogenomes of the five *Bipolaris* species examined, particularly between *B. maydis* and *B. cookei*, with a range of 27.62% ((max-min)/min) (Fig. 1). Previous studies had shown that fungal mitogenome expansion/contraction is closely related to the accumulation of repetitive sequences, plasmid-derived genes, intergenic regions, and intron dynamics (Boussau et al. 2011; Chen et al. 2021; Wang et al. 2020; Zubaer et al. 2018). In the present study, using the mitogenome of *B. maydis* as a reference, we found that intron regions contributed the most to mitochondrial expansion of *B. zeicola*, *B. oryzae*, *B. sorokiniana* and *B. cookei* with 106.09%, 129.64%, 118.10%, and 90.35%, respectively (Additional file 2: Fig. S1). This was consistent with previous findings that intron regions play a major role in the process of fungal mitogenome size changes (Li et al. 2019; Li et al. 2020). Similarly, using the mitogenome of *B. cookei* as a reference, we found that the intronic region contributed the most to this contraction process in *B. maydis* and *B. sorokiniana*, but the intergenic region contributed more than the intronic region to this contraction process in *B. zeicola* and *B. oryzae*. These finds also suggested that the role of intergenic regions in mitochondrial size changes was also important. In addition, repetitive sequences also contributed to the expansion/contraction process, albeit much less than intronic regions. No plasmid-derived genes were found in any of the five *Bipolaris* mitogenomes, the result similar to that observed for the mitogenomes of the *Exserohilum* genus (Ma et al. 2022). Overall, the three newly sequenced *Bipolaris* mitogenomes and the other two reported *Bipolaris* mitogenomes in this study would advance our understanding of mitogenome size boundaries and variation in the *Bipolaris* genus (Zaccaron et al. 2017; Zhang et al. 2023; Deng et al. 2019).

### Gene content variation in *Bipolaris* species

The integration of alpha-proteobacteria into pro-eukaryotes in an endosymbiotic manner was believed to be the origin of mitochondrial organelles in eukaryotes (Gray et al. 2001; Muñoz-Gómez et al. 2017). In the process of long-term evolution, some genes in the mitogenome were lost which was a very common phenomenon observed in mitochondrial genome studies of many fungi (Li et al. 2023; Li et al. 2022; Adams et al. 2003; Adams et al. 2002). However, a number of genes retained in the mitogenome, including a core set of PCGs, 2 rRNAs with 5-35 tRNAs (Ma et al. 2022). These genes played important roles in cellular energy metabolism and in response to physical environmental change (Allen 2015). Like the mitogenomes of most fungi, the mitogenomes of the five *Bipolaris* species contained a set of PCGs, but the *atp8* and *atp9* genes were not found. The *atp8* and *atp9* genes exist in many orders of *Dothideomycetes*, but they were not detecting in mitogenomes of all *Pleosporales* species reported to date (Ma et al. 2021; Ma et al. 2022; Song et al. 2020). We speculated that they were likely lost at some point in *Pleosporales* evolution. Meanwhile, the mitogenome core PCGs of the five *Bipolaris* species had a significantly difference in sequence length, GC content, base composition, and codon usages. The effect of variation in these core PCGs on mitochondrial function needed to be further investigated. K2P results indicated that these core PCGs had different evolutionary rates, but all underwent purifying selection in their evolution (Fig. 5). In addition, some non-conserved PCGs with unknown functions still existed in the mitogenomes of the five *Bipolaris* species. Their functions needed to be further addressed, which was of great significance for understanding the origin and function of mitogenomes. Moreover, the size and base composition of rRNA and tRNA genes in mitogenomes of the five *Bipolaris* species showed variation (Additional file 1: Table S3 and Fig. 2). It had been reported that mutations in the tRNA anticodon arm can lead to changes in the specific recognition of mRNA codons, which could impact protein synthesis (Lin et al. 2021; Ding et al. 2019; Giegé et al. 1998). The effect of mutations in tRNA on *Bipolaris* species requires further study.

### Dynamic changes of introns in *cox1* gene of *Pleosporales*

Introns were commonly found in fungal mitogenomes, and their accumulation, movement and degeneration caused intron polymorphisms in different fungal species and affected the organization and size of fungal mitogenomes (Ma et al. 2022; Sosa-Gómez et al. 2009; Xu et al. 2009; Qin et al. 2010). In the present study, we also found that introns were the main factor contributing to the size variation of *Bipolaris* mitogenomes. In addition, further research had found that these introns were mainly classified into groups I and II, with group I introns being relatively abundant in fungal mitogenomes and containing homing endonucleases that might facilitate intron transfer (Li et al. 2023). Previous studies found that the *cox1* gene was usually the host gene with the highest intron content in the mitogenome (Férandon et al. 2010). In this study, we also found that the *cox1* gene had the highest intron content in the 16 *Pleosporales* mitogenome who contained 118 introns, accounting for 31.13% of the total introns. We further assigned introns from the *cox1* gene to pcls, and identical pcls from different species were considered orthologous introns (Additional file 2: Fig. S2). These pcls were ideal tools for studying genetic variation within fungi and responding to species evolutionary relationships (Megarioti et al. 2020; Michel et al. 1995). In this research, we found that the content and type of pcl in *Pleosporales* mitogenomes varied significantly, reflecting frequent intron loss and gain events in *Pleosporales* species (Fig.4). However, this variation appeared to be controlled within certain limits in mitogenomes of five species of the *Bipolaris* genus (Fig. 4). P615, P1057, P1107 and P1262 were detected in all five *Bipolaris* mitogenomes, P731, P867, P1125 and P1296 were missing in only one *Bipolaris* mitogenome, and similar results were observed in the mitogenome of the genus *Exserohilum* (Ma et al. 2022). We speculated that these universal introns might have been inherited from their common ancestor. In addition, of the 16 *Pleosporales* mitogenomes, P372 was detected only in *B. cookei* of the genus *Bipolaris*, while homologous introns were detected in *Basidiomycetes* far from the genus *Bipolaris*, suggesting that potential intron transfer events might have occurred between distant species (Zaccaron et al. 2017). Further sequencing and analysis of additional mitogenomes was required to reveal the potential mechanisms of origin, transfer, and evolution of these introns.

### Gene rearrangement of *Pleosporales* species

Mitochondrial gene arrangements could provide useful information on genetic variation and phylogenetic relationships between species (Li et al. 2018; Li et al. 2021; Zheng et al. 2018). Currently, mitochondrial gene rearrangements in fungi were far less well studied than in animals. Several models had been used to explain mitochondrial gene rearrangements in animals, but a large number of studies had shown that more complex rearrangement mechanisms might exist in fungal mitogenomes (Lavrov et al. 2002; Xia et al. 2016). We found large-scale gene rearrangements in 16 *Pleosporales* mitogenomes, but five mitogenomes of *Bipolaris* species had very conserved gene arrangements, except for gene inversions occurring only between *cox1* and *cox2* genes in the *B. oryzae* mitogenome (Fig. 7). Meanwhile, the gene arrangement of *Bipolaris* mitogenomes was highly consistent with that of *Exserohilum* mitogenomes, and this unique gene arrangement might have originated from their common ancestor. In contrast to the genus *Bipolaris*, a vastly different set of gene arrangements was found in *C. trifolii* of the genus *Curvularia* (Fig. 7). This variation was due to the inversion of gene fragments between *cob* to *nad2*. Previous studies had suggested that the accumulation of repetitive DNA elements in the intergenic region was an important cause of mitochondrial gene rearrangements, but we detected a much lower content of repetitive sequences in *C. trifolii* mitogenome (9.78%) than in members of the *Bipolaris* genus (ranging from 19.21% to 24.67%) (Additional file 1: Table S6). In view of the above, factors other than repetitive sequences might exist in fungal mitogenomes to influence the gene rearrangement process, and mitogenomes of genera *Bipolaris*, *Exserohilum*, and *Curvularia* could be used as a typical example to provide useful information for further exploration of the mechanism of mitochondrial gene rearrangement in fungi.

### Phylogenetic relationships of *Ascomycota* based on mitochondrial genes

The genus *Bipolaris* is a diverse group that is a widely distributed taxa of fungi (Manamgoda et al. 2014; Manamgoda et al. 2012; Manamgoda et al. 2011). *Bipolaris* pathogens were mainly harmful to food crops such as maize, rice and wheat, causing serious disasters for human society (Manamgoda et al. 2014). Accurate classification and identification of *Bipolaris* fungi will help people understand the disease and control the pathogen in a timely manner. However, morphological identification of *Bipolaris* fungi faced problems such as lack of sexual morphology and confusion of asexual phenotypes (Manamgoda et al. 2014; Manamgoda et al. 2011). Mislabelling sequence information in databases had also caused problems in molecular identification of species in *Bipolaris* genus (Bhunjun et al. 2020). Therefore, phylogenetic analyses of fungi of the genus *Bipolaris*, especially pathogenic fungi, using mitogenomes, are of great importance for the accurate classification of the genus *Bipolaris*. In this study, based on the combined mitochondrial gene set and two phylogenetic inference methods, we obtained a good support phylogenetic tree containing 96 species of *Ascomycota* (Additional file 1: Table S8 and Fig. 8). We found that the five species of the *Bipolaris* were clustered together with high support. In addition, the clustering of genera *Bipolaris*, *Exserohilum*, and *Curvularia* in the phylogenetic tree was consistent with the phylogenetic results of the multi-gene dataset (Hernández-Restrepo et al. 2018). Although the gene arrangements of genera *Bipolaris* and *Exserohilum* were highly congruent, *Curvularia* species were closer to *Bipolaris* species in the phylogenetic tree (Fig. 7 and Fig. 8). To summarize, phylogeny results based on the combined mitochondrial gene set suggested that the mitogenome had a promising application for species delimitation within and between closely related genera of the genus *Bipolaris*. Further sequencing of the mitogenomes of species in the genus *Bipolaris* and closely related genera is needed to better understand the phylogenetic relationships and mechanisms of genetic variation within and among closely related genera.

## Conclusions

In this study, the three newly *Bipolaris* mitogenomes (*B. maydis*, *B. zeicola*, and *B. oryzae*) were sequenced, assembled and compared with two reported *Bipolaris* mitogenomes (*B. sorokiniana* and *B. cookei*). Mitogenomes of the five *Bipolaris* species exhibited significant variation in size, with intronic regions contributing the most to mitogenome expansion. We also observed significant variability in gene contents, gene length, base composition, codon usages, rRNAs, and tRNAs in the mitogenomes of the five *Bipolaris* species. Comparative mitogenomic analyses revealed highly consistent gene arrangement between *Bipolaris* and *Exserohilum* species, but significant gene rearrangements were observed between *Bipolaris* and *Curvularia* species. In addition, the core PCGs of the mitogenomes of 16 *Pleosporales* species exhibited varying evolutionary rates, but all of these genes underwent conserved purifying selection throughout evolution. The potential loss, gain, and transfer events of introns were also detected in the *cox1* genes of 16 *Pleosporales* mitogenomes. Finally, phylogenetic analyses based on the combined mitochondrial gene set and two phylogenetic inference methods revealed the five species of the *Bipolaris* were clustered together with high support. Compared to the genus *Exserohilum*, the genera *Curvularia* and *Bipolaris* were more closely related. This study is the first report on the mitogenomes of *B. maydis* and *B. zeicola*, as well as the first comparison of mitogenomes among species within the genus *Bipolaris*. The results of this study will further advance investigations into the population genetics, evolution, and genomics of *Bipolaris* species.

### Supplementary Information

Additional file 1.  

Additional file 2: Fig. S1.The proportion of different genetic compositions and their contribution to mitogenome expansion (above) and contraction (below) in five *Bipolaris* mitogenomes.

Additional file 3: Fig. S2.Insertion sites of different position classes (Pcls) in the coding regions of *cox1* genes of 16 species. Protein sequences encoded by the *cox1* genes of 15 other species were aligned with the *cox1* of* Bipolaris* *cookei*. The Pcls were named according to their insertion sites in the reference *cox1* sequence of *B. cookei*. The symbols ‘+1’ and ‘+2’ refer to the different insertion positions of Pcls within triplet codons: ‘+1’ when between the 1st and 2nd nt of a codon; and ‘+2’ when between the 2nt and 3nd nt of a codon.

Additional file 4: Fig. S3.Collinearity analysis of five *Bipolaris* mitogenomes as generated with Mauve 2.4.0. Homologous regions between different species were represented by the same color blocks and connected by the same color lines. BC: *Bipolaris cookei*, BZ: *B. zeicola*, BO: *B. oryzae*, BS: *B. sorokiniana*, BM: *B. maydis*.

## Data Availability

All data generated or analyzed during this study are included in this published article [and its Additional files].

## Abbreviations

Mitogenome: Mitochondrial genome
PCG: Protein-coding gene
Pcls: Position classes
*Ks*: Synonymous substitution rates
*Ka*: Nonsynonymous substitution rates
BI: Bayesian inference
ML: Maximum Likelihood
ITS: internal transcribed spacer
*tef1*: translation elongation factor-1 alpha
GAPDH: glyceraldehyde-3-phosphate dehydrogenase
