# Supplementary figures and images for: The complete mitochondrial genomes of five critical phytopathogenic Bipolaris species: features, evolution, and phylogeny
Source: IMA Fungus. 2024 Jun 11;15:15. doi: 10.1186/s43008-024-00149-6 (PMC11167856; doi:10.1186/s43008-024-00149-6)

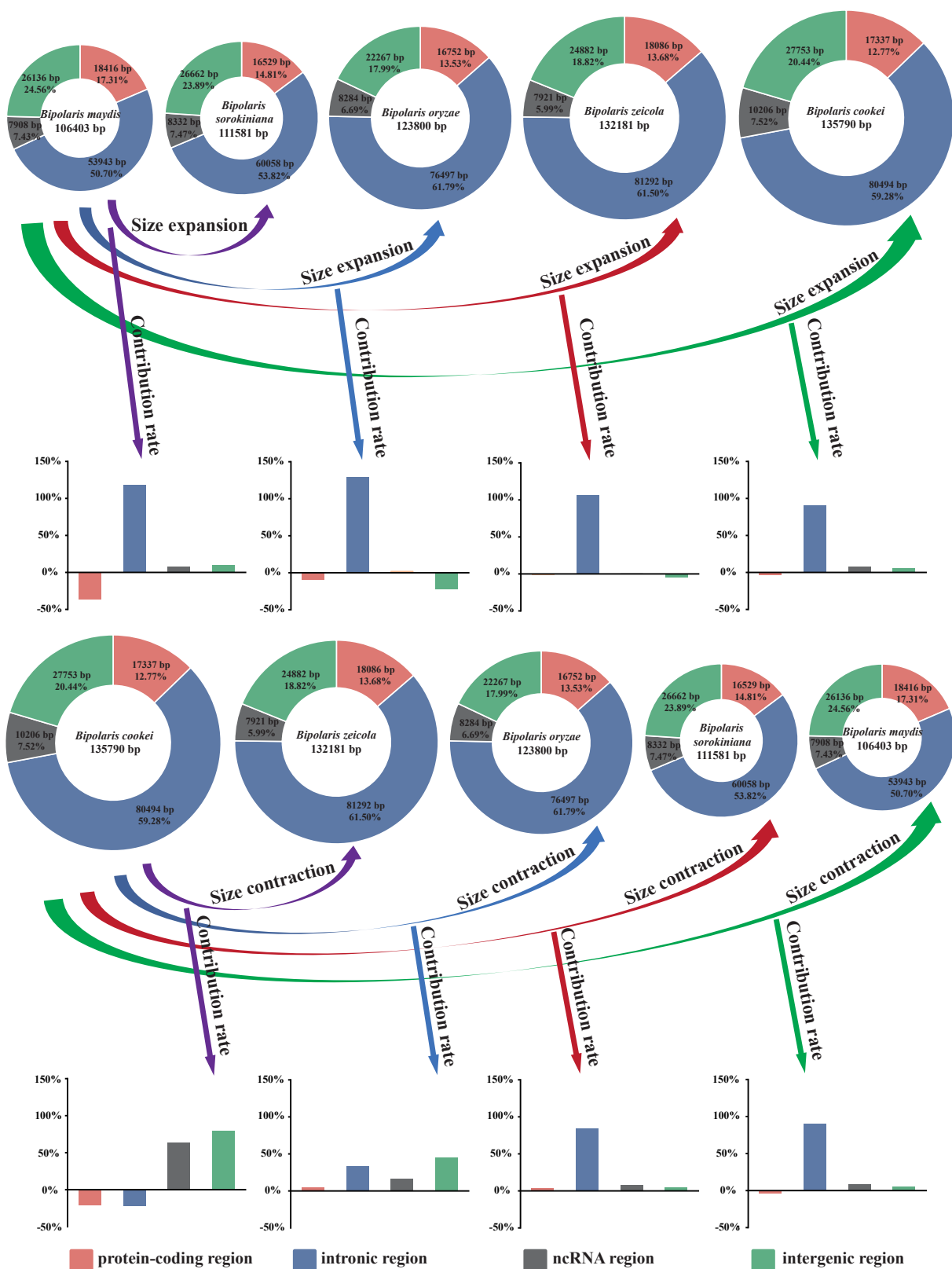

Supplement: Supplementary file 2 — Additional file 2: Fig. S1.The proportion of different genetic compositions and their contribution to mitogenome expansion (above) and contraction (below) in five Bipolaris mitogenomes. [file 43008_2024_149_MOESM2_ESM.pdf]

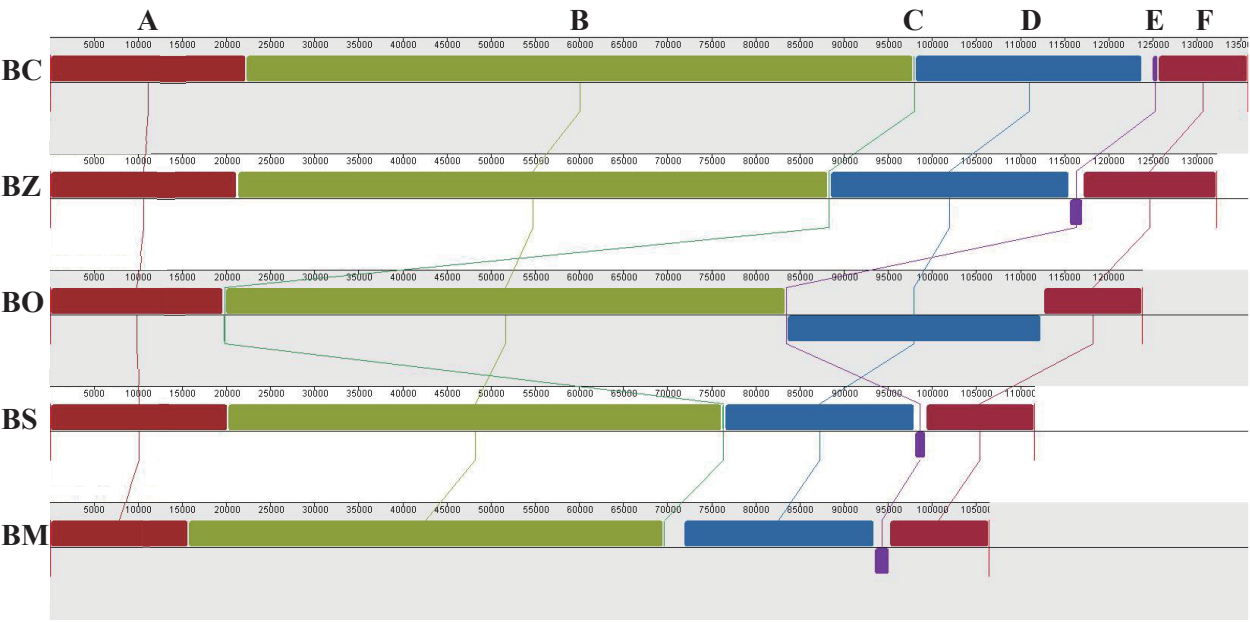

Supplement: Supplementary file 4 — Additional file 4: Fig. S3.Collinearity analysis of five Bipolaris mitogenomes as generated with Mauve 2.4.0. Homologous regions between different species were represented by the same color blocks and connected by the same color lines. BC: Bipolaris cookei, BZ: B. zeicola, BO: B. oryzae, BS: B. sorokiniana, BM: B. maydis. [file 43008_2024_149_MOESM4_ESM.pdf]
