# Supplementary material for: The complete mitochondrial genomes of five critical phytopathogenic Bipolaris species: features, evolution, and phylogeny
Source: IMA Fungus. 2024 Jun 11;15:15. doi: 10.1186/s43008-024-00149-6 (PMC11167856; doi:10.1186/s43008-024-00149-6)
Supplement: Supplementary file 3 — Additional file 3: Fig. S2.Insertion sites of different position classes (Pcls) in the coding regions of cox1 genes of 16 species. Protein sequences encoded by the cox1 genes of 15 other species were aligned with the cox1 of Bipolaris cookei. The Pcls were named according to their insertion sites in the reference cox1 sequence of B. cookei. The symbols ‘+1’ and ‘+2’ refer to the different insertion positions of Pcls within triplet codons: ‘+1’ when between the 1st and 2nd nt of a codon; and ‘+2’ when between the 2nt and 3nd nt of a codon. [file 43008_2024_149_MOESM3_ESM.pdf]

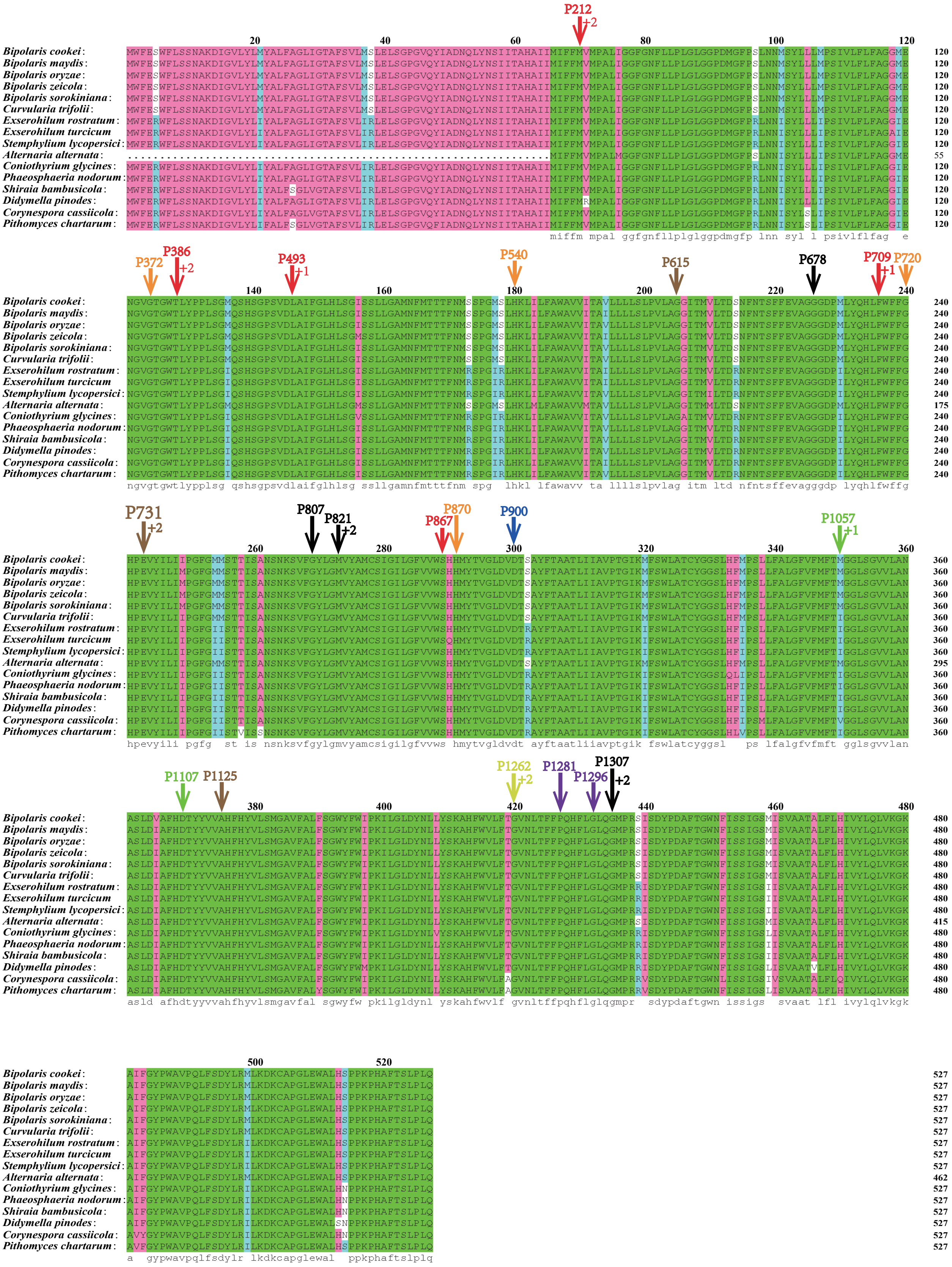
P212 +2
P372
P386 +2
P493 +1
P540
P615
P678
P709 +1
P720
P731 +2
P807
P821 +2
P867
P870
P900
P1057 +1
P1107
P1125
P1262 +2
P1281
P1296
P1307 +2
Bipolaris cookei
Bipolaris maydis
Bipolaris oryzae
Bipolaris zeicola
Bipolaris sorokiniana
Curvularia trifolii
Exserohilum rostratum
Exserohilum turcicum
Stemphylium lycopersici
Alternaria alternata
Coniothyrium glycines
Phaeosphaeria nodorum
Shiraia bambusicola
Didymella pinodes
Corynespora cassiicola
Pithomyces chartarum
